# The Value of Expert Testimony in Medico-Legal Cases from the Medical Standpoint*Read at the thirtieth annual meeting of the Medical Association of Central New York at Buffalo, October 19, 1897.

**Published:** 1898-01

**Authors:** A. W. Henckell

**Affiliations:** Rochester, New York


### THE VALUE OF EXPERT TESTIMONY IN MEDICO-LEGAL CASES FROM THE MEDICAL STANDPOINT.*

BY A. W. HENCKELL, M. D., ROCHESTER, NEW YORK.

I have selected the subject, "The Value of Expert Testimony in Medico-Legal Cases," not for the purpose of giving a lecture on medical jurisprudence, but with the object of bringing to your attention a few salient points gathered from some recent criminal cases relative to expert testimony. I cite the cases of which my paper will largely consist for the purpose of proving that medico-legal testimony, as given before our courts to-day, is little less than a farce and is bringing dishonor and chagrin upon the experts representing our profession, and, worse, contempt for the profession itself.

Wharton defines the "expert" tersely as "such men who are experienced in a specialty."

The present form of examining experts in insanity cases I will not dwell upon. The mode of examining experts is, usually, in the form of a hypothetical question, put and paid for by the side calling the experts; in other words, the professional man testifies to certain opinions (which a man can change at will) for the benefit of the highest bidder. Has the medical fraternity reached so low a level as to forget the duties it owes to truth and veracity?

The expert should be called for the purpose of assisting the law in the obtaining of justice for the prisoner, to assist the court and elucidate certain points to the jury. The hypothetical question has many objections urged against it. Hornblower says:

> It is claimed, and with much truth, that the hypothetical question assumes as proved whatever the counsel putting the question has endeavored to prove, and combines insignificant with important circumstances and alleged facts, supported by slight and perhaps worthless testimony, with other facts of which the proof is strong and convincing, while omitting still other facts of equal or greater importance which may be overwhelmingly established by the other side.

Expert testimony based upon such one-sided hypothetical questions is almost, of necessity, favorable to the questioner, and the true remedy is that already adopted by the laws of this State in insanity cases—the appointment of a commission of experts.

The primary object of expert testimony is not to prove opinions but facts, in the shape of rules of science or art, generally recognized by those who are especially instructed in such art or science.

Lord Campbell stated, in the Tracy Peerage case, that "skilled witnesses come with such a bias on their minds, to

---

*Read at the thirtieth annual meeting of the Medical Association of Central New York at Buffalo, October 19, 1897.

support the cause in which they embark, that hardly any weight should be given to their evidence."

Judge Davis, of the Supreme Court of Maine, stated: "If there is any kind of testimony that is not only of no value but often worse than that, it is, in my judgment, that of medical experts."

A certain justice of the Supreme Court of our own State, in speaking of witnesses, once said: "There are three kinds of witnesses—liars, damn liars and 'experts.'"

From this it will appear how necessary it is for medical experts to free themselves from all bias and swear only to the truth, the whole truth and nothing but the truth, without fear or favor.

These criticisms are not always merited, and, if medical opinions were free from bias, such opinions would be of inestimable value, as they are in Germany and France, where the expert becomes an official and a component part of the judicial system. The objection to the expert in this country is that parties pay and retain their own experts. The question of the compensation of the medical expert is one which has attracted the attention of the profession. Although most writers on medical jurisprudence make mention of the fact that they should, or must, receive remuneration, the question, I believe, has never been decided in this State. I have the opinion of one of the justices of the Supreme Court to the effect that the question has never been judicially settled. He stated, as a reason, that no physician would risk being fined for contempt, but would rather answer the question.

In the recent Carlyle Harris trial, the most eminent experts in the State(and out of it) were called to give evidence. These men testified to facts, so-called, but they were diametrically opposed to each other. Of what value was this testimony in the obtaining of justice?

Again, in a recent murder trial in New York City, experts were on the stand, day after day, for the purpose of deciding the question whether or not the presence of human blood could be positively determined; but here again it was of no value to either side.

Some here will recall a case of alleged rape that occurred in the town of Gates, Monroe county. The Humane Society, through its agent, tried to convict—I can say with perfect frankness, he tried to convict—justice was not thought of, even by the officers of the Humane Society. At the hearing before the justice of the peace a female physician was put upon the stand as an expert and testified that she was of the opinion that this little ten-year old girl had been assaulted; she was willing to say this on the ground that the hymen had been ruptured, the vagina large and roomy. It would seem impossible that a physician, at this late day, could swear that the absence of the hymen was proof positive of unchastity in the individual, but such was the case. In this case the Humane Society was over-anxious to convict. Their own expert, Dr. Moore, maintained that the examination revealed that the girl had not been assaulted, and on that score advised the Humane Society's attorney to refuse me an examination, I having been called by the defense.

Why, in the spirit of fairness, did this society not endeavor to assist in freeing the man from this accusation after their own expert's opinion had been expressed? Why was not Dr. Moore called to the stand? Why did not the prosecuting attorney ask for the discharge of the prisoner on account of the lack of evidence? But none of it. He insisted that the accused be held for the grand jury; that he be lodged in jail, obliged to undergo the anxiety of a trial, because this woman "expert" swore that the girl had been misused. Was expert testimony of value here? Did it accomplish the ends of justice? No; decidedly not. It was a disgrace to the society prosecuting and unfortunate that the profession could not have forced its testimony. It was not until this man was brought to trial that one of the plans I advocated was tested—namely, the appointment of a commission. Three experts, appointed by the court, unbiased to the prejudice of the prisoner or the financial considerations from either side, examined the girl, and their finding caused the immediate discharge of the prisoner.

I maintain that this, or a similar plan, should be adopted in all legal proceedings. That is the practice in France and Germany, and few indeed are the objections urged against it.

In the Benham case the medical expert testimony was again brought into ridicule, to such an extent that the jury practically ignored the testimony of all the experts. The reason for this was very apparent, in that the jurors were not familiar with chemistry and its intricate technicalities. It is even asserted that whilst an eminent expert was giving his testimony a number of jurors fell into temporary somnolency.

Again, in the Luetgert case, now pending, we find the experts at war with each other and exposed to the ridicule of the public. The aptness of some expert witnesses in murder trials is exemplified in this trial. One of the leading esteologists for the defense on being shown the skull of a mammal pronounced it as being the skull of a dog, describing in minutest detail its complete structure. After finishing his explanation the prosecuting attorney informed him that it was the skull of a monkey. Recognizing the pit he had fallen into he humorously remarked it was probably a monkey-faced dog.

Considering the various phases of expert testimony one can readily see that a remedy must be sought for and speedily put into practice if the honored status of the medical profession is to be maintained. In my opinion there are two ways out of the difficulty. The first, the appointment of a commission by the court (I have already alluded to it in the early part of my paper); the other, the appointment of a commission of experts to receive the contentions of the experts of each side, and they to decide and present their findings as facts to the jury and not to be subject to review. In my opinion the latter plan is freer from criticism.—*Buffalo Medical Journal.*

